# A Longitudinal PET/MRI Study of Colony-Stimulating Factor 1 Receptor–Mediated Microglia Depletion in Experimental Stroke

**DOI:** 10.2967/jnumed.121.262279

**Published:** 2022-03

**Authors:** Cristina Barca, Amanda J. Kiliaan, Claudia Foray, Lydia Wachsmuth, Sven Hermann, Cornelius Faber, Michael Schäfers, Maximilian Wiesmann, Andreas H. Jacobs, Bastian Zinnhardt

**Affiliations:** 1 European Institute for Molecular Imaging, University of Münster, Münster, Germany;; 2 Department of Medical Imaging/Anatomy, Radboud University Medical Center, Radboud, The Netherlands;; 3 Clinic of Radiology, Translational Research Imaging Center, University Hospital Münster, Münster, Germany;; 4 Department of Nuclear Medicine, University Hospital Münster, Münster, Germany;; 5 Department of Geriatrics and Neurology, Johanniter Hospital, Bonn, Germany; and; 6 Biomarkers and Translational Technologies, Pharma Research and Early Development, F. Hoffmann-La Roche Ltd., Basel, Switzerland

**Keywords:** colony stimulating factor-1 receptor, microglia, stroke, ^18^F-DPA-714, MRI

## Abstract

Microglia-induced neuroinflammation after stroke contributes to the exacerbation of postischemic damage but also supports neurorestorative events. Longitudinal molecular imaging of microglia-targeted therapies will support the assessment of target engagement, therapy efficacy, and deciphering of the mode of action. We investigated the effects of chronic colony-stimulating factor 1 receptor (CSF-1R) inhibitor–mediated microglia depletion on translocator protein (TSPO)–dependent neuroinflammation and cerebrovascular parameters using PET/MRI. **Methods:** Forty C57BL/6 mice underwent a 30-min transient occlusion of the middle cerebral artery and were randomly assigned to either a control group or a group treated with CSF-1R inhibitor (PLX5622). Eight mice per group were used for *N,N*-diethyl-2-(2-(4-(2-^18^F-fluoroethoxy) phenyl)5,7dimethylpyrazolo[1, 5a]pyrimidin-3-yl)acetamide (^18^F-DPA-714) (TSPO) PET imaging on days 7, 14, 21, and 30 after ischemia and behavioral tests before and after surgery. An extra group of 8 mice underwent MRI, including T2-weighted (infarct), perfusion-weighted (cerebral blood flow), and diffusion-weighted (water diffusion, cellular density) sequences, on days 1, 3, 7, 14, 21, and 30. Ex vivo analysis (immunoreactivity, gene expression) was performed to characterize the inflammatory environment. **Results:** We demonstrated that long-term CSF-1R inhibition transiently decreased the TSPO PET signal within the infarct. Residual TSPO activity was partly due to a potentially resistant Iba-1–positive cell populations with low CSF-1R and transmembrane 119 expression. The decrease in selected pro- and antiinflammatory marker expression suggested an apparent global dampening of the neuroinflammatory response. Furthermore, the temporal changes in the MRI parameters highlighted treatment-induced effects on reperfusion and tissue homeostasis, associated with impaired motor function at late stages. **Conclusion:** Longitudinal TSPO PET/MRI allows the assessment of target engagement and optimization of drug efficiency. PLX5622 has promising immunomodulatory effects, and the optimal therapeutic time window for its application needs to be defined.

## Visual Abstract

**Figure:**
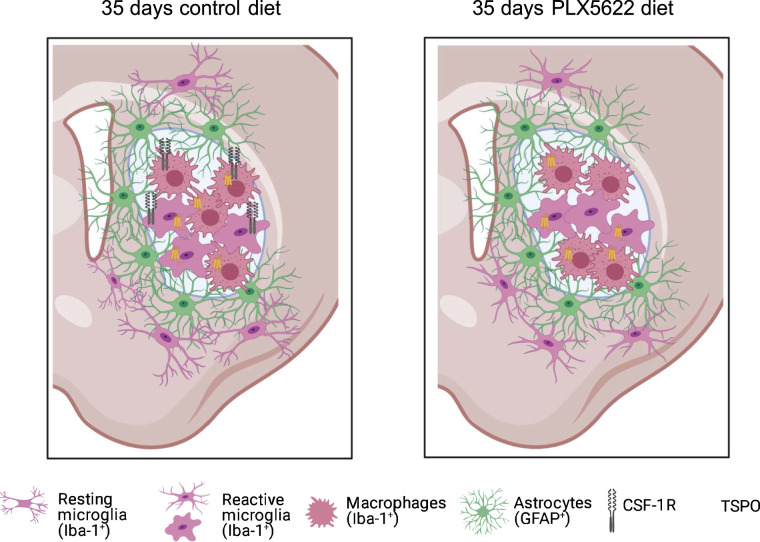

Neuroinflammation and microglial activity are key contributors to stroke pathogenesis (*1*). Microglia are essential in brain immunosurveillance, homeostasis monitoring, and synaptic landscape shaping at steady state. In pathological conditions such as cerebral ischemia, microglia undergo transcriptomic alterations resulting in an early protective antiinflammatory phenotype within the first week, quickly switching to a detrimental proinflammatory phenotype at around days 10–14 after ischemia, thus worsening inflammation and causing tissue damage (*2*). Therefore, modulating microglia reactivity may offer a new approach in the treatment of cerebral ischemia.

The colony-stimulating factor 1 receptor (CSF-1R) is a well-described tyrosine kinase involved in differentiation, proliferation, and survival of resident microglial cells but also perivascular and bone marrow-derived macrophages and other cell types (osteoclasts, dendritic cells) (*3*). Recently, the selective CSF-1R inhibitor PLX5622 was reported as an efficient treatment to deplete microglial cells in healthy (*4*,*5*) and pathological (*6*,*7*) conditions. In a healthy brain, CSF-1R inhibition leads to 50% depletion of Iba-1–positive microglial cells after 3 d and near-complete depletion within 7 d, with few side effects. In an inflammatory environment, PLX5622 treatment led to beneficial outcomes: it efficiently depleted microglial cells, shifted the remaining cells toward a more antiinflammatory phenotype, and alleviated or improved symptoms (*6*–*10*). The use of PLX5622 offers the opportunity to assess the contribution of microglial activity to the neuroinflammatory reaction and potentially represents a new therapeutic approach in stroke. The first preclinical preconditioning studies have shown PLX5622 treatment to worsen disease outcomes within the first days (*4*,*11*,*12*), increasing infarct size and promoting primary inflammation. However, none of these studies assessed its potential effect as a long-term treatment. Thus, there is interest in performing a longitudinal study using various imaging, functional, and ex vivo assessments to characterize changes induced by the CSF-1R inhibitor PLX5622 on stroke-relevant parameters such as inflammation, on cerebrovascular parameters, and on functional outcomes.

We performed such a longitudinal study, using PET/MRI to noninvasively monitor therapy response, including *N,N*-diethyl-2-(2-(4-(2-^18^F-fluoroethoxy)phenyl)5,7dimethylpyrazolo[1,5a]pyrimidin-3-yl) acetamide (^18^F-DPA-714) (translocator protein [TSPO], neuroinflammation) PET imaging, T_2_-weighted (T_2_w, lesion) MRI, diffusion-weighted (water diffusion) MRI, and perfusion-weighted (cerebral blood flow [CBF]) MRI. In addition, we performed behavioral tests, including open-field (locomotion), pole test (global motor functions), grip test (limb strength), and rotarod test (coordination), to assess sensorimotor functions.

We hypothesized that CSF-1R inhibition might represent a new therapeutic intervention in modulating microglia-induced postischemic inflammation. Long-term CSF-1R inhibition may reduce microglial activity and therefore decrease the expression of inflammatory markers and improve recovery. The potential impact of CSF-1R inhibition may be noninvasively assessed by combining an extensive multiparameter PET/MRI paradigm with behavioral tests and ex vivo analysis.

## MATERIALS AND METHODS

### Study Approval

All experiments were conducted in accordance with the German Law on the Care and Use of Laboratory Animals and approved by the Landesamt für Natur, Umwelt, und Verbraucherschutz of North Rhine-Westphalia according to the ARRIVE guidelines (https://www.nc3rs.org.uk/arrive-guidelines).

### Study

In total, 48 male C57BL6/6J mice 3–4 mo old were housed under a standard 12-h:12-h light:dark cycle with free access to food and water.

**FIGURE 1.**
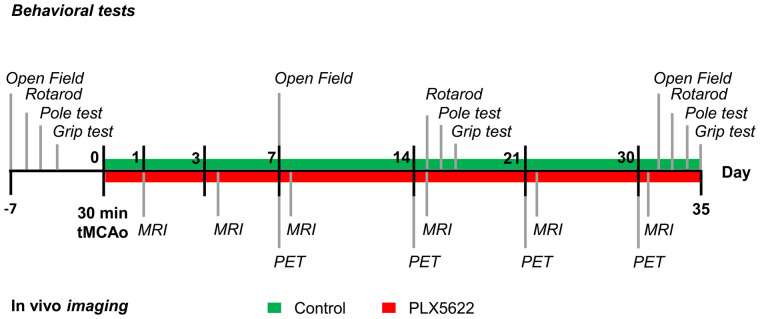
Study design. tMCAo = transient middle cerebral artery occlusion.

Forty mice underwent a 30-min transient occlusion of the middle cerebral artery on day 0 (Fig. 1); right after surgery, they were randomized—by a person other than the experimenters—into either a control group or a group receiving dietary PLX5622 treatment. The experimenters did not know the group assignment. All mice underwent T_2_w MRI on day 1 to select animals on the basis of the infarct size. Exclusion criteria were a lack of reperfusion (<50% baseline CBF recovery) as assessed by laser Doppler, an infarct exceeding the striatal and cortical regions, and extreme weight loss (>20% of the initial body weight). The dropout rate was 4%.

Eight wild-type (nonstroke) mice were used for additionally ex vivo analysis on day 35 after ischemia. Those mice received either a control diet (*n* = 4) or the PLX5622 diet (*n* = 8) for 35 d and were killed for gene expression analysis. The animal groups and numbers are detailed in Supplemental Table 1 (supplemental materials are available at http://jnm.snmjournals.org).

### Experimental Design

Eight mice per group were used for in vivo PET imaging and behavioral tests. ^18^F-DPA-714 PET imaging was conducted on days 7, 14, 21, and 30 after surgery. Another 2 groups of 8 mice underwent MRI on days 1, 3, 7, 14, 21, and 30 after surgery, including T_2_w, diffusion-weighted, and perfusion-weighted sequences. PET/MRI sessions were performed 12 h apart. All animals were continuously anesthetized during surgery and image acquisition. The mice underwent behavioral tests before and at around days 7, 14, and 30 after surgery to assess motor function recovery using open-field, rotarod, pole, and grip testing.

On day 35, all animals were anesthetized and killed by transcardiac perfusion. The brains were harvested for immunohistochemistry and immunofluorescence analysis to assess the long-term effects of PLX5622 therapy on the neuroinflammatory response. In addition, 4 groups of 4 mice that did not undergo imaging were added for real-time quantitative polymerase chain reaction analysis of tissues harvested on day 35 after ischemia.

The experimental time line is summarized in Figure 1.

### Surgery

The mice underwent a 30-min transient occlusion of the middle cerebral artery (MCA) of the right hemisphere using an intraluminal occlusion model as previously described, with minor modifications (*13*). Briefly, the mice received 0.04 mg of fentanyl (RotexMedica) and 4 mg of midazolam (Ratiopharm) per 1 g of body weight before surgery. Transient focal cerebral ischemia was induced by introducing a silicone-coated 7–0 monofilament (diameter with coating, 0.19 ± 0.01 mm) (Doccol Corp.), which was withdrawn after 30 min. The mice received a subcutaneous injection of buprenorphine after surgery (0.05–0.1 mg/kg) (Indivior).

### Treatment

PLX5622 was provided by Plexxikon Inc. and formulated in AIN-76A standard chow by Research Diets Inc. at 1,200 ppm chow. Body weight was tracked as an index of food intake (Supplemental Fig. 1).

### ^18^F-DPA-714 PET/CT Imaging

^18^F-DPA-714 was prepared as previously described with more than 99% radiochemical purity (*14*). PET imaging was performed with a high-resolution small-animal PET scanner (32-module quadHIDAC [Oxford Positron Systems Ltd.]; spatial resolution, <1 mm in full width at half maximum) (*15*).

The ^18^F-DPA-714 PET scan was acquired from 45 to 65 min after injection of 12.1 ± 2.0 MBq (specific activity, 40–80 GBq/μmol). Immediately afterward, a CT scan was acquired using an Inveon CT scanner (Siemens Medical Solutions; spatial resolution, 80 μm).

### MR Neuroimaging

T_2_w MRI (repetition time, 7,700 ms; effective echo time, 100 ms; RARE [rapid acquisition with relaxation enhancement] factor, 30; matrix, 192 × 192; averages, 8), diffusion-weighted MRI (repetition time, 2,500 ms; echo time, 31.30 ms; b-values, 100, 200, 400, 600, 800, 1,000, 1,200, 1,600, and 2,400 s/mm^2^; matrix, 128 × 128; averages, 8), and perfusion-weighted MRI (repetition time, 10,000 ms; echo time, 5.01 ms; slices, 1; matrix, 64 × 64) were performed using a 9.4-T small-animal MRI scanner (Biospec 94/20; Bruker Biospin GmbH) with a 2-mm surface coil (Bruker) as previously described (*16*).

### Image Analysis

Image data were analyzed using the in-house–developed software MEDgical, allowing analysis of multidimensional, multiscale biomedical image data, as previously described (*16*).

All MR images were manually superimposed on the corresponding PET/CT images of the same animal. An atlas-based thresholding approach was used to delineate the infarct volume on day 1 after ischemia (*17*). Regional ^18^F-DPA-714 uptake (percentage injected dose [%ID]/cm^3^) was assessed within the T_2_w MRI-based infarct and the contralateral atlas-based striatum.

Similarly, the T_2_w MRI-based infarct and its mirrored image were superimposed on the apparent diffusion coefficient (ADC) and arterial spin labeling (ASL) maps to assess water ADC and CBF, respectively. The infarct-to-contralateral ratio was also calculated to account for intraindividual variability.

### Immunoreactivity and Quantification

To assess the TSPO cellular source, we performed immunofluorescent TSPO/ionized calcium binding adaptor molecule 1 (Iba-1) and TSPO/glial fibrillary acidic protein (GFAP) costaining as previously described (*16*). Additionally, Iba-1/CSF-1R and Iba-1/transmembrane 119 (TMEM119) staining were performed to further characterize the Iba-1–positive cell population.

For image validation and semiquantification, we performed TSPO, Iba-1, and GFAP immunostaining as previously described (*16*). Antibodies are reported in Supplemental Table 2.

Sections were viewed with a combined fluorescent–light microscope (Eclipse NI-E; Nikon), and images were analyzed using ImageJ software (version 1.51j; National Institutes of Health).

### Behavioral Tests

Open-field, grip, rotarod, and pole testing was performed to evaluate the therapeutic effects of CSF-1R inhibition on motor function recovery during the postischemic period, as previously detailed (*16*). The 4 behavioral tests were performed before and after surgery, as indicated in Figure 1.

### Gene Expression

Total RNA was isolated from snap-frozen half-brain tissues (RNeasy mini kit; Qiagen), and DNase I treatment (Roche) was used to avoid contamination from genomic DNA. One microgram of total RNA was reverse-transcribed into first-strand complementary DNA using the Transcriptor First Strand complementary DNA synthesis kit (Roche).

The forward and reverse primer sequences (Sigma-Aldrich) are reported in Supplemental Table 3. Real-time quantitative polymerase chain reaction testing was performed using the Rotor-Gene SYBR Green Master mix with the Rotor-Gene Q device (Qiagen). Relative gene expression was assessed using the ΔΔCt method, with *Gapdh* (Biomol Gmbh) as a housekeeping gene.

### Statistics

Statistical analysis was performed using SigmaPlot (Systat Software GmbH). All data were tested for normality and equal variance using the Shapiro–Wilk and Brown–Forsythe tests, respectively. Nonparametric tests were used when assumptions of normality or equal variance were not met. In all statistical tests, differences were considered significant when *P* values were less than 0.05.

Repeated measures (RM) ANOVA, followed by the Holm–Šídák post hoc test, was performed for multiple comparisons. Data were expressed as mean ± SEM. For gene expression data analysis, RM ANOVA with Tukey multiple comparisons was used. For percentage of stained area, data were displayed as box plots and reported as mean ± SD.

The sample sizes were calculated a priori during the animal ethics dossier application. They were based on effect size (*P* = 0.05; statistical power, 0.80), mortality rates, and a previous stroke study (*18*) in which we investigated the therapeutic effect of a dietary approach on brain inflammation as assessed by an ^18^F-DPA-714 PET imaging study on ischemic mice. We set the minimal detectable difference in means to 0.2 and the expected SD of residuals to 0.1.

## RESULTS

For all stroke mice, T_2_w MRI infarct volume significantly decreased over time (*P* < 0.001). No treatment effect was observed (*P* = 0.54) (Supplemental Fig. 2).

**FIGURE 2.**
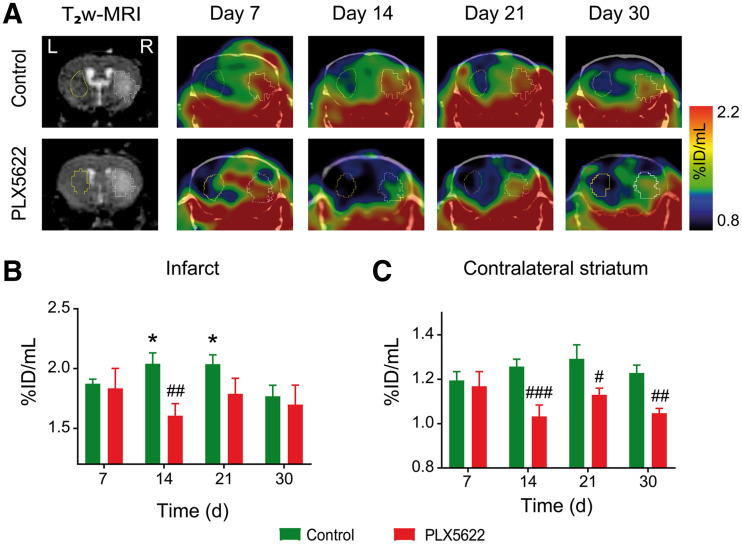
(A) Representative ^18^F-DPA-714 (TSPO) PET/CT images and corresponding day 1 T_2_w MR image. (B and C) Quantification of ^18^F-DPA-714 uptake (%ID/mL) within infarct (B) and contralateral striatum (C). *P < 0.05 vs. day 30. #*P* < 0.05 vs. treatment. ##*P* < 0.01 vs. treatment. ###*P* < 0.005 vs. treatment.

First, we performed longitudinal ^18^F-DPA-714 PET/CT on both control and PLX5622-treated mice to assess the potential immunomodulatory effect of PLX5622 (Fig. 2A). Individual data are shown in Supplemental Fig. 3. Two-way RM ANOVA indicated treatment (*P* = 0.014; power, 0.80) and treatment × time (*P* = 0.011) effects but no time effect (*P* = 0.412) on tracer uptake within the infarct (Fig. 2B). A significant reduction in tracer uptake was observed in PLX5622-treated mice on day 14 after ischemia compared with control mice (control, 2.04 ± 0.09 %ID/cm^3^; PLX5622, 1.60 ± 0.10 %ID/cm^3^; *P* = 0.009). Additionally, two-way RM ANOVA indicated a significant effect of treatment (*P* = 0.003; power, 0.89) but not of time (*P* = 0.227) or time × treatment (*P* = 0.084) in the contralateral striatum (Fig. 2C). ^18^F-DPA-714 uptake was significantly decreased in PLX5622-treated mice compared with control mice on days 14 (*P* < 0.005), 21 (*P* = 0.013), and 30 (*P* = 0.006) after ischemia, indicative of a permanent drug effect on TSPO levels in noninfarcted tissue. ^18^F-DPA-714 uptake correlated with TSPO immunoreactivity on brain tissue collected on day 35 after ischemia (Supplemental Figs. 4–5, *R*^2^ = 0.91). No treatment effect was observed on ^18^F-DPA-714 uptake in spleen (*P* = 0.20) or manubrium sternebra (*P* = 0.16) over time (Supplemental Fig. 6).

**FIGURE 3.**
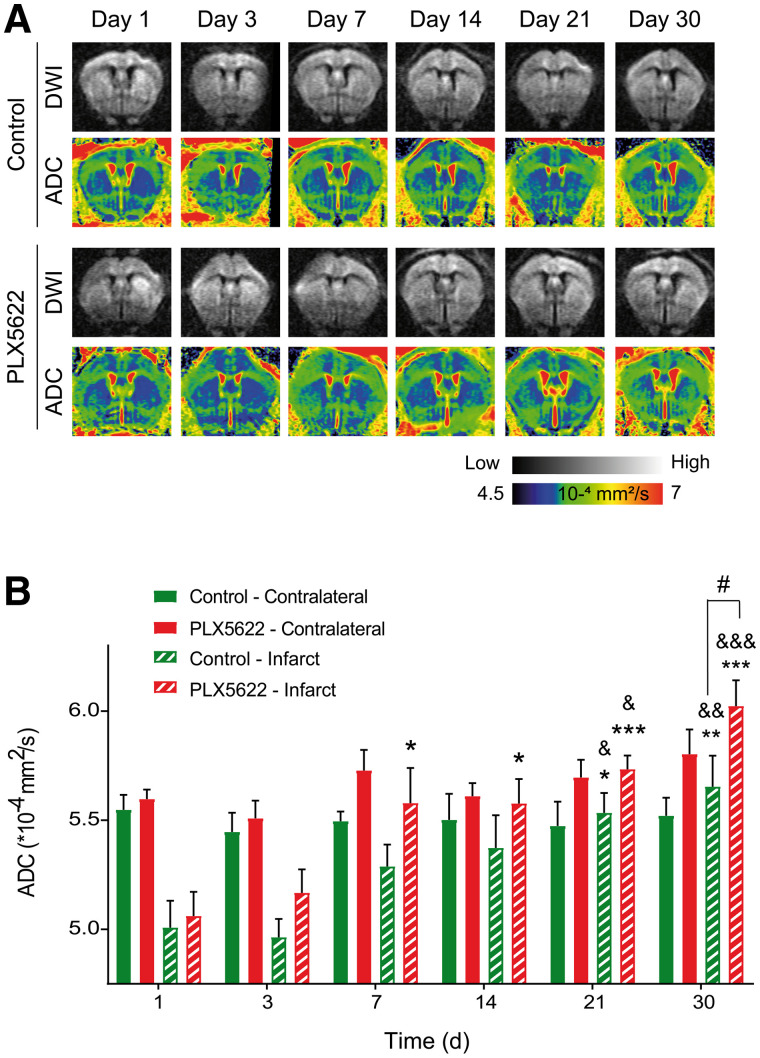
(A) Representative diffusion-weighted images (for b = 2,400 s/mm^2^) and respective ADC maps. (B) Time course of ADCs, indicating homeostatic imbalance within infarct at day 30 with treatment. DWI = diffusion-weighted imaging. *P < 0.05 vs. day 1. **P < 0.01 vs. day 1. ***P < 0.005 vs. day 1. #*P* < 0.05 vs. treatment. &*P* < 0.05 vs. day 3. &&*P* < 0.01 vs. day 3. &&&*P* < 0.005 vs. day 3.

Furthermore, we tracked therapy response on 2 stroke-associated MRI parameters, ADC and CBF. Representative diffusion-weighted images and the respective ADC maps from control and PLX5622-treated mice are shown in Figure 3A. RM ANOVA indicated main effects of treatment (*P* = 0.011; power, 0.55) and time (*P* < 0.001) but not of time × treatment (*P* = 0.2) on ADC within the infarct. In control mice, ADC significantly increased on days 21 (*P* = 0.022) and 30 (*P* = 0.006) compared with day 1 after ischemia. Similarly, ADC significantly increased on days 21 (*P* = 0.10) and 30 (*P* = 0.003) compared with day 3 (Fig. 3B). In PLX5622-treated mice, the ADC within the infarct was significantly increased on days 7 (*P* = 0.011), 14 (*P* = 0.10), 21 (*P* < 0.001), and 30 (*P* < 0.001) compared with day 1. Additionally, ADCs were significantly increased on days 21 (*P* = 0.016) and 30 (*P* < 0.001) compared with day 3. A treatment effect was observed on day 30 after ischemia, with the ADC of PLX5622-treated mice within the infarct being significantly higher than that of control mice (*P* = 0.028) (Fig. 3B). No treatment effect was observed on ADCs in the contralateral region (*P* = 0.10). The intraindividual follow-up of the infarct-to-contralateral ratio indicated comparable temporal recovery between groups (Supplemental Fig. 7).

**FIGURE 4.**
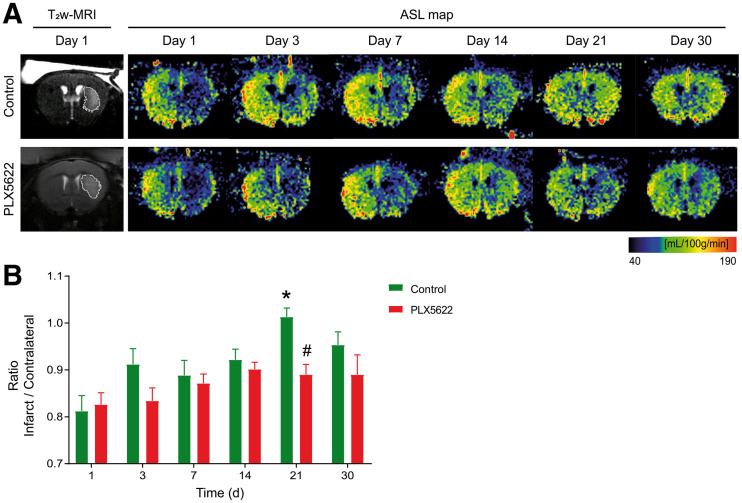
(A) Representative arterial spin labeling maps and corresponding day 1 T_2_w MR image. (B) PLX5622-treated mice showing significantly lower infarct-to-contralateral ratio than in control mice on day 21, indicative of impaired tissue reperfusion within infarct. ASL = arterial spin labeling. *P < 0.05 vs. day 1. #*P* < 0.05 vs. treatment.

Representative ASL maps from control and PLX5622-treated mice are shown in Figure 4A. To account for intraindividual variability, we reported the temporal dynamic of the infarct-to-contralateral ratio for both groups (Fig. 4B). Two-way RM ANOVA indicated a significant effect of time (*P* = 0.045), treatment (*P* = 0.042; power, 0.49), and time × treatment (*P* = 0.019). In control mice, the infarct-to-contralateral ratio significantly increased on day 21 compared with day 1 (*P* = 0.028), whereas PLX5622-treated mice did not show any significant difference over time (*P* = 0.15). A therapy effect was observed on day 21, with control mice showing an increased infarct-to-contralateral ratio (1.00 ± 0.03) compared with PLX5622-treated mice (0.89 ± 0.03) (*P* = 0.048). Mean CBFs are reported in Supplemental Fig. 8.

We quantified the number of Iba-1–positive cells within the infarct, at the periphery of the infarct, and in the contralateral striatum of both groups as an index of therapy efficiency (Supplemental Fig. 9A). Two-way RM ANOVA indicated treatment (*P* < 0.001) and region (*P* < 0.001) effects. Treatment effects were detected at the periphery (*P* = 0.035) and contralateral side (*P* < 0.001), with the percentage of Iba-1–positive area being higher in control mice than in PLX5622-treated mice (Supplemental Fig. 9B).

**FIGURE 5.**
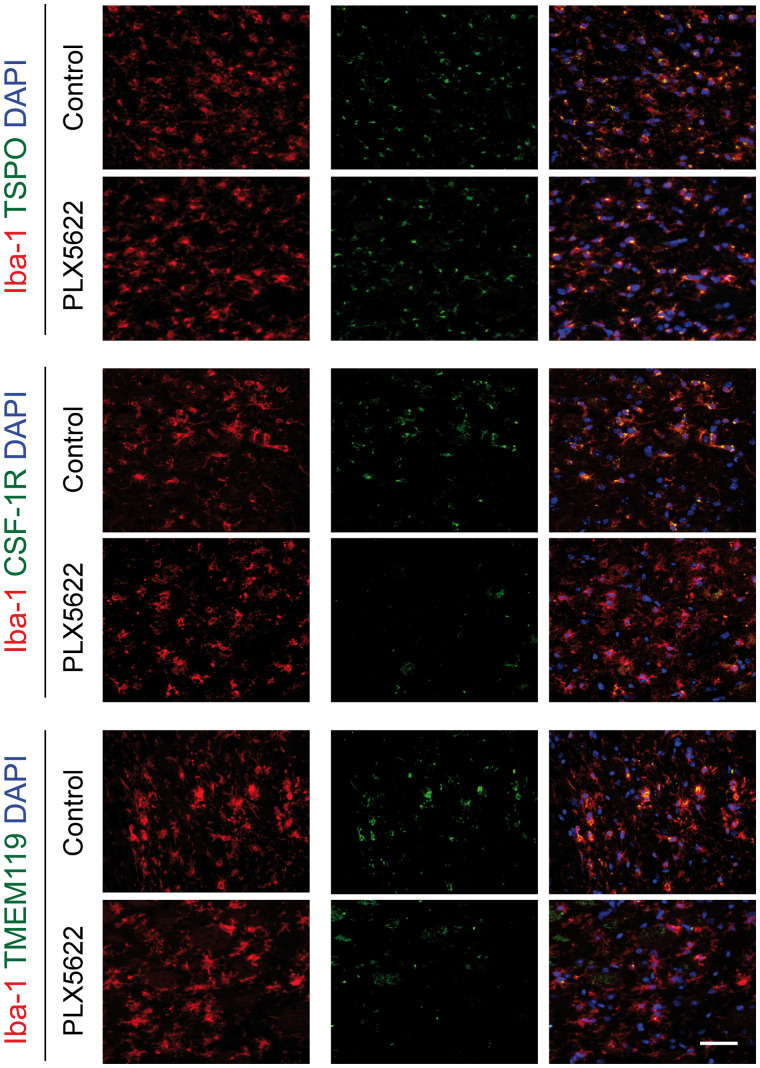
Immunofluorescent staining for TSPO, CSF-1R, and TMEM119 in Iba-1–positive cell population within infarct in both control and PLX5622-treated mice on day 35 after ischemia. DAPI = 4′,6-diamidino-2-phenylindole. Scale bar = 15 μm.

Qualitative assessment of Iba-1–positive cells indicated that microglia and macrophages showed elongated thin processes in control mice, indicative of a resting state, whereas they showed shorter, thicker processes with large soma in PLX5622-treated mice, indicative of a reactive state (Fig. 5; Supplemental Fig. 9B). Additionally, the number of ramifications at the periphery and contralateral striatum was higher in control than in PLX5622-treated mice.

Increased astrogliosis was observed in the contralateral striatum of PLX5622-treated mice (Supplemental Fig. 10).

**FIGURE 6.**
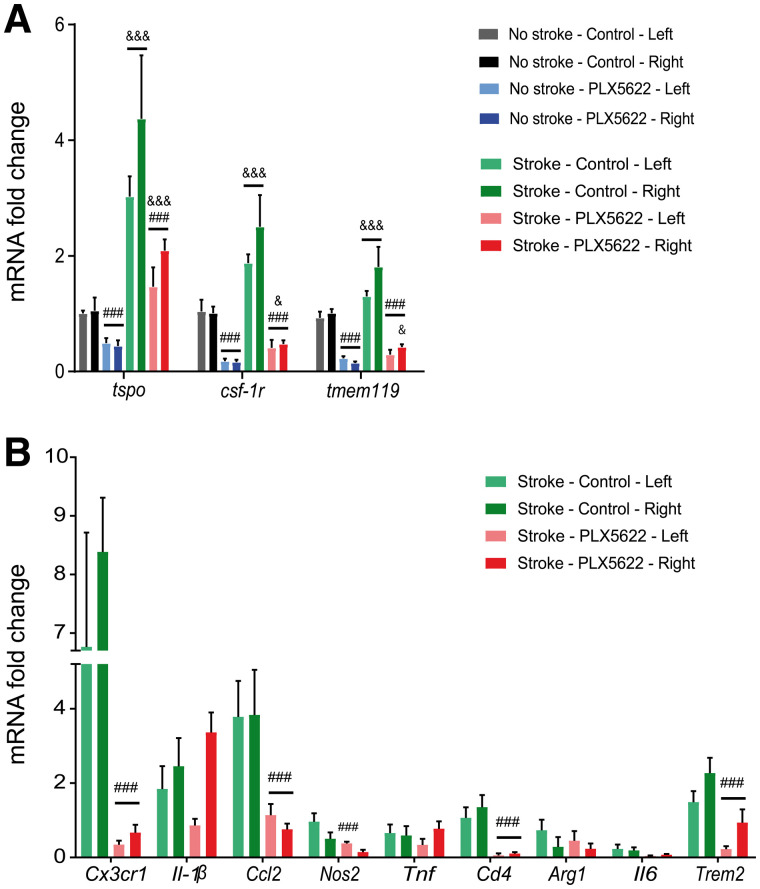
(A) Significant decrease in *tspo, csf-1r,* and *tmem119* gene expression with treatment in both wild-type and stroke animals. (B) Efficient downregulation of gene transcription for several proinflammatory, antiinflammatory, and phagocytosis-relevant genes on CSF-1R inhibition. mRNA = messenger RNA. ###*P* < 0.005 vs. treatment. &*P* < 0.05 vs. wild-type mice. &&&*P* < 0.005 vs. wild-type mice.

We further characterized the Iba-1–positive cell population by immunofluorescence. Brain slices from day 35 were costained with TSPO, CSF-1R, and TMEM119 (Fig. 5). Both control and PLX5622-treated mice showed a strong population of Iba-1–positive TSPO-positive cells within the infarct, indicating that microglia and macrophages are a TSPO cellular source whereas most of the glial fibrillary acidic protein–positive cells (astrocytes) were TSPO-negative (Supplemental Fig. 11). However, we observed many Iba-1–positive CSF-1R–positive and Iba-1–positive TMEM119–positive cells in control mice, whereas none or few were observed in PLX5622-treated mice. Immunoreactivity was cross-validated by looking at messenger RNA expression of *tspo, csf-1r,* and *tmem119* in wild-type and stroke animals (Fig. 6A). *Tspo, csf-1r,* and *tmem119* were downregulated after 35 d of treatment (*P* < 0.005) in both wild-type and stroke mice, with no significant difference between the left and right hemispheres.

Long-term PLX5622 treatment globally reduced proinflammatory *Cx3cr1* and *Ccl2* and antiinflammatory *Cd4* expression (Fig. 6B). *Nos2* was downregulated in the contralateral hemisphere but was unaffected in the infarct hemisphere. On the other hand, PLX5622 treatment did not change messenger RNA expression of proinflammatory *Il-1β* and *tnf* and antiinflammatory *Arg1* and *Il-6* (*P* > 0.05). Additionally, PLX5622 significantly downregulated the phagocytosis-related *trem2* gene marker in both hemispheres.

Motor deficit recovery was used as an index of treatment efficiency (Supplemental Figs. 12–15). Overall, 3 of the 4 tests indicated a treatment effect on day 30 after ischemia. Although no significant effect was observed on traveled distance and velocity in the open test (*P* = 0.75), PLX5622-treated mice walked a shorter distance in the rotarod test (*P* = 0.023), showed less forelimb strength in the grip test (*P* = 0.028), and moved more slowly during the pole test (*P* = 0.041) than control mice.

## DISCUSSION

CSF-1R inhibition–induced microglia depletion represents a valuable tool to investigate the contribution of microglia activity to postischemic brain injury. For the first time, our study showed the potential of ^18^F-DPA-714 PET imaging to track the immunomodulatory effect of PLX5622 in stroke. We found that CSF-1R inhibition transiently decreased radiotracer uptake within the infarct whereas a sustained decrease was observed in the contralateral healthy tissue. Ex vivo characterization suggested that one of the major cellular sources of TSPO expression was a potentially therapy-resistant Iba-1–positive cell population. Further characterization indicated that those cells were mostly CSF-1R–negative and TMEM119-negative, in line with the significant decrease in *csf-1r* and *tmem119* gene expression. We concluded that PLX5622 efficiently inhibited CSF-1R and affected at least the main population of Iba-1–positive CSF-1R–positive cells, including (TMEM119-positive) resident microglia, and dampened the expression of some inflammatory markers. Moreover, the contribution from peripheral immune subpopulations to the immune cell pool may be reduced, as indicated by the decrease in *cx3cr1, ccl2,* and *cd4* gene expression. Long-term CSF-1R inhibition also affected homeostatic balance and tissue reperfusion, albeit transient, as indicated by our MRI data.

Altogether, our study highlighted a PLX5622 immunomodulatory effect in stroke, which can be noninvasively assessed by ^18^F-DPA-714 PET imaging. PLX5622 affected subpopulations of microglial cells but did not reduce the total number of Iba-1–positive cells. MRI data supported vascular and homeostasis impairment after long-term treatment.

TSPO PET is currently the most studied method for spatial measurement and visualization of neuroinflammation, with a bench-to-bed translational value, and other non-TSPO tracers are currently being developed (*19*). TSPO is markedly upregulated by immune cells such as inner brain glial cells (microglia, astrocytes) and peripheral immune cells during inflammatory conditions, making TSPO a suitable biomarker for tracking neuroinflammation and immune cell activation. Because of the improved bioavailability, specificity, and signal-to-noise ratio of ^18^F-DPA-714 compared with other TSPO tracers, ^18^F-DPA-714 was used in target validation studies on preclinical and clinical stroke and to track the response to immunomodulatory treatment (*20*). In humans, image quantification requires patient stratification because the rs6971 polymorphism causes inter -  and  intraindividual   variability   in binding affinity. In rodents, the established temporal dynamics of ^18^F-DPA-714 in stroke indicate that the number of  CD11b–positive TSPO–positive/Iba-1–positive TSPO–positive cells peaks at around days 11–14, correlating with the peak of radiotracer uptake (*21*,*22*). Here, we showed a transient decrease in TSPO expression within the infarct in PLX5622-treated mice but a continuous decrease in healthy tissue from day 14 after ischemia. Therefore, we hypothesized that the decrease in ^18^F-DPA-714 signal at around day 14 was potentially caused by the depletion of microglia and macrophages.

Ex vivo characterization revealed a mixed population of Iba-1–positive/CSF-1R–negative and Iba-1–positive/TMEM119-negative cells within the infarct, with no significant therapy effect on the total number of Iba-1–positive cells. This result indicated that PLX5622 may affect subpopulations of Iba-1–positive cells, including resident homeostatic TMEM119-positive microglial cells (*23*), in line with the significantly decreased *csf-1r, tmem119,* and *Cx3cr1* (myeloid lineage) gene expression. As previously reported (*24*), PLX5622-treated mice also displayed reactive or dystrophic Iba-1–positive cell morphology in perilesional areas compared with control mice, indicative of a nonresting state. Altogether, changes in protein or gene expression and morphology might indicate a shift in cell functionality, as supported by our gene expression data.

Additionally, the decrease in *cx3cr1* (myeloid lineage), *ccl2* (monocytes), and *cd4* (T cells) gene expression indicated that the peripheral response may be dampened. Similarly, Lei et al. observed a significant reduction in monocytes, dendritic cells, and T cells of the myeloid or lymphoid compartment in bone marrow, spleen, and blood (*25*) after long-term treatment. Therefore, further research must consider the role of invading immune cell in the disease phenotype, focusing on (Iba-1–positive) perivascular, dendritic, and monocytic cells that were not fully depleted with treatment.

Our MRI data indicated that long-term PLX5622 treatment promotes homeostatic imbalance and impairs vascular integrity, which might partly explain the late functional decline. We propose that the absence of CSF-1R–positive cells, including parenchymal and perivascular microglial cells, might transiently impair reperfusion because of increased vascular leakage (*26*) or atrophy (*27*).

The therapeutic effects of dietary interventions depend on food intake. The mice showed a significant body weight loss within the first days, indicating that a therapeutic drug concentration may have been reached only after a few days; this delay may potentially have acted as a confounding factor.

## CONCLUSION

We demonstrated that long-term CSF-1R inhibition during the postischemic phase represents an attractive pharmacologic tool allowing timely modulation of the inflammatory microenvironment. The potential of PLX5622 as an immunomodulatory treatment is supported by our longitudinal DPA-714 PET/MRI data. We demonstrated its therapy effect on global TSPO-related inflammation but also detrimental side effects on reperfusion and homeostasis. Further investigations are needed to determine the treatment time window that maximizes therapeutic effect and avoids a potential negative impact. Noninvasive imaging techniques allowing intraindividual and longitudinal assessments support the identification of specific treatment time intervals.

## DISCLOSURE

This work was partly funded by the Horizon 2020 Programme under grant agreement 675417 (PET3D), the “Cells-in-Motion” Cluster of Excellence (DFG EXC1003-CiM), the Herbert-Worch-Stiftung, and the Interdisciplinary Center for Clinical Research (IZKF core unit PIX), Münster. No other potential conflict of interest relevant to this article was reported.

## Notes

KEY POINTS **QUESTION:** Is PET/MRI a valuable tool to assess therapeutic microglia modulation after cerebral ischemia? **PERTINENT FINDINGS:** Long-term PLX5622 treatment has immunomodulatory and detrimental effects on stroke as assessed by multiparametric PET/MRI. **IMPLICATIONS FOR PATIENT CARE:** PET/MRI may allow tracking of target engagement and of the efficiency of microglia-targeting therapies and refinement of the treatment algorithm.
